# Overexpression of Epcam and CD133 Correlates with Poor Prognosis in Dual-phenotype Hepatocellular Carcinoma

**DOI:** 10.7150/jca.41090

**Published:** 2020-03-05

**Authors:** Jie Zhang, Ya-Peng Qi, Ning Ma, Fei Lu, Weng-Feng Gong, Bin Chen, Liang Ma, Jian-Hong Zhong, Bang-De Xiang, Le-Qun Li

**Affiliations:** 1 Department of Hepatobiliary Surgery, Guangxi Medical University Cancer Hospital, Guangxi, China; 2 Graduate School of Health Science, Suzuka University of Medical Science, Suzuka, Japan

**Keywords:** dual-phenotype hepatocellular carcinoma, prognosis, Cancer stem cells, CD133, EpCAM

## Abstract

**Background**: Dual-phenotype hepatocellular carcinoma (DPHCC) is associated with high rate of post-operative recurrence and low rate of survival, which may reflect the post-operative persistence of cancer stem cells (CSCs). Here we explored the potential correlation between DPHCC and expression of CSCs markers.

**Methods**: In this retrospective study, we included 19 patients with DPHCC and 61 patients with non-DPHCC treated in 2015 by liver resection. Paraffin-embedded tumor tissue specimens were analyzed using immunohistochemistry as well as immunofluorescence double-staining. Rates of recurrence-free survival and overall survival were compared between the two groups using the Kaplan-Meier method, and expression of the CSC markers CD133, CD90, and EpCAM were compared using real-time quantitative PCR and western blotting.

**Results**: Overall survival rates were significantly lower for patients with DPHCC than patients with non-DPHCC at 1 year (78.9% vs 93.4%), 2 years (52.6% vs 72.1%), and 3 years (42.1% vs 67.2%) (*P* = 0.019). Multivariate Cox proportional hazard modeling identified CK19 positivity (*P* = 0.016) and multiple nodules (P = 0.023) as independent predictors of poor recurrence-free survival. Independent predictors of poor overall survival were CK19 positivity (P = 0.032), Barcelona Clinic Liver Cancer stage C (*P* = 0.025) and carbohydrate antigen 19-9 (CA19-9) >37 ng/ml (*P* = 0.016). Expression of CD133 and EpCAM mRNA and protein were significantly higher in DPHCC tissue than non-DPHCC tissue, while CD90 expression was similar between the groups.

**Conclusions**: These results suggest that DPHCC is associated with significantly lower overall survival than non-DPHCC, and that the poor prognosis among DPHCC patients may be related to the presence of CSCs expressing CD133 and EpCAM.

## Introduction

Hepatocellular carcinoma (HCC) is one of the most common malignancies in the world and is the third most frequent cause of cancer mortality^1^. Surgical resection is the most effective way to treat HCC^2^,^3^. However, the 5-year recurrence rate is up to 70% after hepatectomy, indicating the need for further work to understand this cancer better 4. HCC occurs as various pathological subtypes, which may differ in prognosis^5^,^6^. Dual-phenotype HCC (DPHCC) is a newly described HCC subtype characterized by the expression of biomarkers for HCC and intrahepatic cholangiocarcinoma. DPHCC accounts for approximately 10% of all cases of HCC^7^, and it shows greater malignancy and invasiveness than non- DPHCC subtypes^8^. The mechanisms behind DPHCC onset are poorly understood.

Positive expression of cytokeratin 19 (CK19) is one of the typical characteristics of DPHCC. CK19 is believed to be a a marker of cancer stem cells (CSCs)^9^,^10^, which show strong proliferation and self-renewal in tumor tissues and can differentiate along several pathways. Moreover, CSCs appear to contribute greatly to tumor formation, development and maintenance^11^. CSCs have been identified in liver cancer tissues and cell lines^12^-^14^, raising the question of how such cells contribute to initial HCC and recurrence. The particularly poor prognosis associated with DPHCC may indicate that, even after resection, sufficient CSCs remain to cause recurrence.

This study compared the prognosis of patients with DPHCC or non-DPHCC after curative hepatectomy. We also examined whether expression of CSCs markers correlated with poor prognosis of DPHCC patients.

## Methods

The study protocol was approved by the Research Ethics Review Board of the Guangxi Medical University Cancer Hospital. Prior to hepatic resection, all patients gave written informed consent for their anonymized data to be analyzed and published for research purposes.

### Patients and tissue samples

This retrospective study analyzed medical records of patients with HCC, all of whom underwent curative hepatic resection as initial treatment at the Guangxi Medical University Cancer Hospital between January 2015 and November 2015. Tumor stage was determined according to the guidelines of the American Association for the Study of Liver Diseases(AASLD)^15^. Patients were included if they met the following criteria: (1) HCC was diagnosed by standardized pathological parameters; (2) Child-Pugh functional liver status was A or B; and (3) initial treatment was curative resection, which was defined as removal of all tumor lesions based on macroscopic inspection and negative histology resection margin, absence of residual tumor or portal tumor thromboses in postoperative imaging, and decrease of alpha-fetoprotein levels to normal within 2 months after surgery. Patients were excluded if they had any other malignancies, distant metastasis, lymph node involvement, or macrovascular invasion. They were also excluded if no paraffin-embedded tissues from them had been archived. A total of 80 patients met the inclusion criteria.

Either paraffin-embedded or frozen tissues were available for 47 of these 80 patients (13 with DPHCC, 34 with non-DPHCC) for western blotting and PCR analyses as described below. The specimens had been archived in the Biobank of the Guangxi Medical University Cancer Hospital.

### Follow-up

All patients were followed up at 1 month after surgery, then every 3 months for the rest of the first year, and every 6 months thereafter. Follow-up visits included physical examination, liver function tests, serum alpha-fetoprotein measurement, abdominal ultrasonography and computed tomography or magnetic resonance imaging. Recurrence-free survival and overall survival were calculated as the time from the date of surgery until detection of recurrent tumors or until the date of the last follow-up. Follow-up was conducted until April 2019.

### Diagnosis of DPHCC

In this study, DPHCC was diagnosed when all the following pathology criteria were satisfied: (1) more than 15% of tumor cells were strongly positive for at least one hepatocyte marker, such as hepatocyte-specific antigen (Hep Par 1), which exhibited mainly a diffuse distribution; (2) more than 15% of tumor cells were strongly positive simultaneously for at least one hepatocyte marker and at least one cholangiocyte marker, such as CK19. A patient was not diagnosed with DPHCC if his or her tissue contained HCC and intrahepatic cholangiocarcinoma elements, regardless of whether there were transitional zones between the elements, or if his or her tissue showed non-overlapping expression of hepatocyte and cholangiocyte markers.

### Immunohistochemistry

Serial sections (4 µm thick) were prepared from 10% formalin-fixed, paraffin-embedded tumor tissue blocks. Sections were rinsed with phosphate-buffered saline, treated with 0.3% hydrogen peroxide for 15 min, and blocked with 10% goat serum for 1 h. Primary antibodies against Hep Par-1 (ab190706, Abcam, UK) or CK19 (ab52625, Abcam) were incubated overnight at 4 ℃. Sections were washed again with phosphate-buffered saline and incubated for 1 h at 37 ℃ with goat anti-rabbit or -mouse secondary antibody (SP-9002; ZSGB-BIO, China). Sections were incubated at room temperature for 15 min with horseradish peroxidase TMB solution (SP-9002, ZSGB-BIO, China), followed by 3,3-diaminobenzidine for 5 min. Slides were counterstained with hematoxylin (G1080, Solarbio, China) for 8 min and dehydrated through a graded alcohol series. Sections were sealed with Permount^TM^ Mounting Medium before microscopy observation.

### Immunofluorescence double-staining

Serial sections (4 µm thick) were cut from 10% formalin-fixed, paraffin-embedded tumor tissue blocks followed by deparaffinization, rehydration and heat-induced epitope retrieval. Sections were simultaneously incubated with primary antibodies against Hep Par-1 and CK19. Then, sections were stained for 1 h at 37 ℃ in the dark with TRITC-conjugated goat anti-rabbit secondary antibody (111-026-045; Jackson ImmunoResearch, USA) and FITC-conjugated goat anti-mouse secondary antibody (115-096-003; Jackson ImmunoResearch). Sections were counterstained for 2 min with 4',6-diamindino-2-phenylindole (C0065; Solarbio, China) to label nuclei, and analyzed under a fluorescence microscope (BX51, Olympus, Japan).

### Real-time quantitative PCR (RT-qPCR)

Total RNA was extracted from tumor tissues using Trizol (Tiangen Biotech, Beijing, China). Complementary DNA was generated using the PrimeScript™ reverse transcription kit (Vazyme, China) and PCR amplification was performed with the SYBR® Premix Ex Taq™ kit (Vazyme) according to the manufacturer's instructions. PCR reactions were analyzed using the qTOWER2.2 System (Analytik Jena, Germany). All primers were synthesized by Shanghai Biological Engineering Co. (Shanghai, China) (Table 1).

### Western blotting

Total protein was isolated from tumor tissues using RIPA (R0010; Solarbio, China), and protein concentration was measured using the BCA protein assay kit (Beyotime, Shanghai, China). Samples were electrophoresed using 10% SDS-PAGE, then proteins were transferred to PVDF membranes (Bio-Rad, Hercules, CA, USA). Membranes were blocked in skim milk and incubated overnight at 4 ℃ with rabbit primary antibodies against CD90, CD133 or epithelial cell adhesion molecule (EpCAM, 1:3000; Abcam). Next, membranes were incubated with horseradish peroxidase-conjugated anti-rabbit IgG antibodies (1:2000; SE134, Solarbio, China) at room temperature for 2 h. Immunoreactive bands were visualized using enhanced chemiluminescence and quantified using Image Pro Plus version 6. β-tubulin was used as an internal protein reference.

### Statistical analysis

Statistical analyses were carried out using SPSS 22.0 (IBM, USA). Statistical significance was defined as P < 0.05. Inter-group differences in categorical data were assessed for significance using the chi-squared test (2-sided) or Fisher's exact test, as appropriate. The Kaplan-Meier method was used to calculate recurrence-free survival and overall survival rates, and inter-group differences in survival curves were assessed using the log-rank test. Multivariate analysis based on Cox proportional hazard regression modeling was used to identify independent predictors of recurrence-free survival and overall survival rates in HCC patients after liver resection.

## Results

During the study period, 298 patients underwent curative liver resection for HCC. However, 218 patients (73.2%) were excluded because CK19 expression in histopathology sections was unknown or no paraffin tissue specimens were available (n = 152, 51.0%); because other procedures, such as local ablation therapy, ethanol injection or transarterial chemoembolization, had been applied prior to liver resection (n = 12, 4.0%); or because patients were lost to follow-up (n = 54, 18.1%). In the end, 80 patients were included in the study.

### Clinicopathological characteristics

Clinicopathological characteristics of the included patients are summarized in Table 2. Although there were three times as many patients in the non-DPHCC group as in the DPHCC group (61 vs. 19), the two groups showed no significant differences in sociodemographic or clinical parameters.

### Risk factors for poor prognosis in HCC

Several risk factors significantly influenced HCC recurrence (Table 3), including CK19 positivity (*P* = 0.007), multiple nodules (*P*=0.005), hepatitis B surface antigen positivity (*P*=0.033) and carbohydrate antigen 19-9 (CA19-9) >37ng/ml (*P*=0.015). Independent risk factors of HCC recurrence were CK19 positivity (HR 1.867, 95%CI 1.124-3.102, *P* = 0.016) and multiple nodules (HR 1.868, 95%CI 1.089-3.205, *P* = 0.023).

Postoperative mortality of patients with HCC was influenced by the following risk factors (Table 3): DPHCC (*P* = 0.023), CK19 positivity (*P* = 0.006), Barcelona Clinic Liver Cancer (BCLC) stage C (*P* = 0.027), multiple nodules (*P* = 0.024), serum albumin <35 g/L (*P* = 0.046) and CA19-9 >37 ng/ml (*P* = 0.017). Further analysis identified the following independent predictors of postoperative morbidity: CK19 positivity (HR 2.213, 95%CI 1.070-4.580, *P* = 0.032), BCLC stage C (HR 2.061, 95%CI 1.093-3.888, *P* = 0.025) and CA199 >37 ng/ml (HR 2.222, 95%CI 1.164-4.242, *P* = 0.016).

### Correlation between DPHCC and CSC marker expression

Levels of CD90 (THY1), CD133 and EpCAM mRNA were measured in tumor tissues from 13 patients with DPHCC and 34 patients with non- DPHCC subtypes (Figure 2). DPHCC was associated with significantly higher levels of CD133 mRNA (*P* = 0.013) and EpCAM mRNA (*P* = 0.006). In contrast, the two patient groups showed similar levels of CD90 mRNA (*P* = 0.441). These mRNA results were mirrored at the protein level.

### Comparison of recurrence-free and overall survival between DPHCC and HCC patients

**Figure 1:**
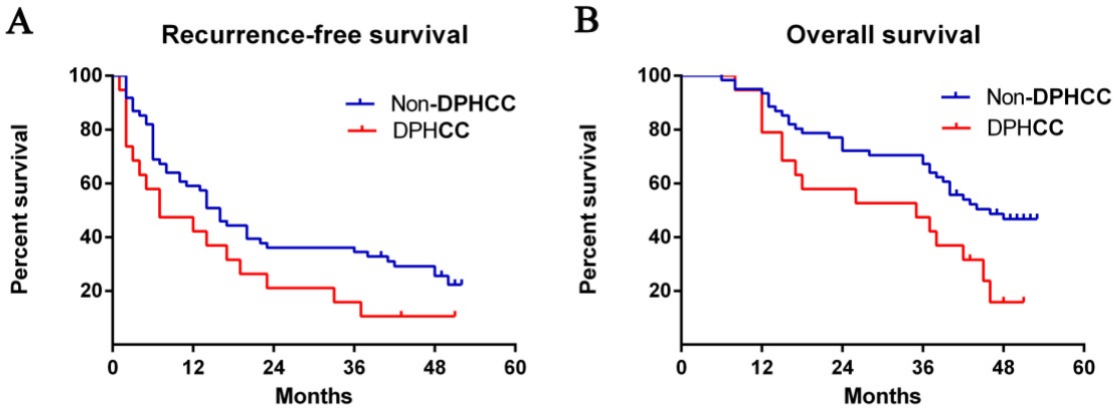
** Postoperative survival rates.** The Kaplan-Meier method was used to calculate **(A)** recurrence-free survival (log rank, P = 0.058) and **(B)** overall survival curves between patients with DPHCC and non-DPHCC (log rank, P = 0.029). Abbreviations: DPHCC, dual-phenotype hepatocellular carcinoma.

**Figure 2:**
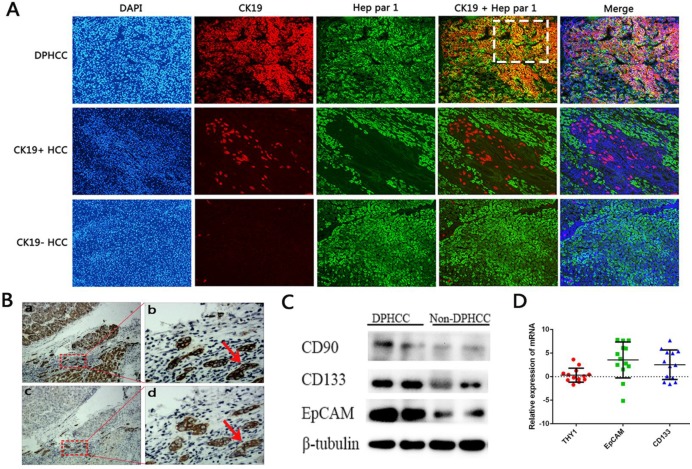
** Expression of cancer stem cell markers. (A)** Immunofluorescence double-staining of hepatic tumor tissues from patients with DPHCC or non-DPHCC for the cholangiocytic marker CK19 (red) and hepatocyte marker Hep Par-1 (green). Patients with non-DPHCC were subdivided into those negative or positive for CK19 expression. The cells in the white dotted box are dual-phenotype cells. **(B)** Immunohistochemical staining of DPHCC tumor tissue for Hep Par 1 and CK19 from patients with HCC. Panels a and c are consecutive slices shown at ×40 magnification, while b and d are zoomed-in views (×200 magnification) of the boxed areas. Cells indicated by red arrows fulfill the DPHCC diagnostic criteria. The cells indicated by the red arrow are dual-phenotype cells. (C) Western blot showing protein levels of CD90, CD133 and EpCAM. (D) Relative levels of mRNAs encoding CD133, EpCAM and CD90 (THY1) as detected by real-time quantitative PCR. Relative expression of mRNA was determined using the log (fold change). Positive values (relative expression of mRNA > 0) were taken to indicate up-regulation.

Median recurrence-free survival was 7 months among patients with DPHCC and 16 months among patients with non-DPHCC subtypes. The DPHCC group showed significantly lower rates of recurrence- free survival at 1 year (42.1% vs 57.4%), 2 years (15.8% vs 36.1%), and 3 years (10.5% vs 34.4%) after surgery (*P* = 0.058, Fig. 1A).

Median overall survival was 35 months among DPHCC patients and 46 months among non-DPHCC patients. Follow-up indicated significantly lower overall survival rates in the DPHCC group at 1 year (78.9% vs 93.4%), 2 years (52.6% vs 72.1%), and 3 years (42.1% vs 63.9%) (*P* = 0.019, Fig. 1B).

## Discussion

The World Health Organization recognizes three types of primary liver cancer: HCC, intrahepatic cholangiocarcinoma, and a combination of hepatocellular carcinoma and cholangiocarcinoma^16^. Few studies have been performed on the combination form, DPHCC, and this research gap is important to address because of the relatively high recurrence rate associated with this subtype.

Our study confirms work by others showing that positive expression of CK19, considered a CSCs marker in HCC^17^, ^18^, is an independent risk factor for decreased overall survival in HCC patients following curative resection^19^-^22^. We further showed here that CK19 can predict poor recurrence-free survival after resection. DPHCC exhibits high invasiveness, which might be closely correlated with the expression of CK19, which mediates transforming growth factor beta (TGFβ)/Smad signaling^23^.

Patients with DPHCC in our cohort showed a lower rate of overall survival than those with non-DPHCC, which is consistent with previous studies^7^, ^8^ and which confirms that DPHCC is a more malignant HCC subtype, even after curative resection. Our study showed that CA19-9 levels > 37 ng/ml are predictors of overall survival in patients with HCC after resection. CA19-9 levels effectively predicted postoperative survival of intrahepatic cholangiocarcinoma patients expressing high levels of CK19^24^. Serum CA19-9 is also a marker of poor prognosis in pancreatic cancer and colorectal carcinoma^25^-^27^.

Although the difference in recurrence-free survival between DPHCC patients and non-DPHCC patients was not statistically significant, survival tended to be lower in patients with DPHCC (P=0.058). Multiple tumor nodules were an independent risk factor for recurrence-free survival in HCC patients, probably owing to the high incidence of microvascular invasion and intrahepatic metastasis in these patients^28^,^29^. Multiple nodules are often accompanied by microsatellite tumors that are invisible to the naked eye; they cannot be accurately removed during surgery, which affects the tumor recurrence rate in HCC patients after hepatectomy.

We found significantly higher levels of EPCAM and CD133 mRNA and protein in DPHCC tissues than in non-DPHCC tissues. Our results are consistent with reports that HCC patients positive for EpCAM or CD133 have poor prognosis, and that EPCAM and CD133 act synergistically in tumors^30^-^32^. EPCAM participates in a Wnt signaling pathway of tumorigenesis^33^,^34^, while CD133+ cancer cells up-regulate interleukin-8 (IL-8) and activate MAP kinase signaling pathways^35^, ^36^. One study showed that IL-8 promoted β-catenin phosphorylation in cancer cells and acted via the Wnt/β-catenin pathway to induce the epithelial-to-mesenchymal transition and migration^37^. Therefore we suspect that EPCAM and CD133 act via Wnt-related pathways to influence HCC, and further research should explore how.

Expression of a third CSCs marker, CD90, was similar between DPHCC and non-DPHCC tissues in our study. CD90 may be involved in HCC only under certain conditions. Although CD90 is a typical marker of CSCs in HCC and high CD90 expression has been linked to higher recurrence rate^38^, ^39^, one study reported that CD90+ CSCs-like cells in the liver may participate only in late-stage liver cancer associated with hepatitis B virus infection^40^.

The results of the present study should be interpreted with caution in light of several limitations, such as lack of long-term overall survival data, small sample and short follow-up. Despite these limitations, our data justify further work exploring the use of immunofluorescence double-staining as a reliable method for diagnosing DPHCC. Our data also suggest a link between high expression of two CSCs markers (EpCAM, CD133) and poor prognosis in DPHCC.

## Supplementary Material

Supplementary methods, results, and figure.

## Figures and Tables

**Table 1:** Real-time quantitative PCR primers

Gene	Forward primer (5' to 3')	Reverse primer (3' to 5')
β-actin	GTCTTCCCCTCCATCGTG	AGGTGTGGTGCCAGATTTTC
CD90 (THY1)	AGAGACTTGGATGAGGAG	CTGAGAATGCTGGAGATG
EPCAM	AGAACCTACTGGATCATCATTGAACTAA	CGCGTTGTGATCTCCTTCTG
CD133	AACCTACAGCATATTCTTCA	AACGAACAGCATTTCTCTCTCAAGA

Abbreviations: β-actin, beta-actin; CD90, cluster of differentiation 90; EPCAM, Epithelial cell adhesion molecule; CD133, cluster of differentiation 133.

**Table 2:** Clinicopathological data of hepatocellular carcinoma patients with DPHCC or non-DPHCC subtypes

Variable	DPHCC	non-DPHCC	P
	N=19	N=61	
**Age, yr**			0.966
≤55	16	49	
>55	3	12	
**Gender**			0.797
Male	17	51	
Female	2	10	
**Liver cirrhosis**			0.058
Yes	3	24	
No	16	37	
**Tumor size, cm**			0.655
≤5.0	7	26	
>5.0	12	35	
**No. of tumors**			0.083
<2	10	45	
≥2	9	16	
**Capsule of tumor**			0.309
Incomplete	9	21	
Complete	10	40	
**Tumor thrombi**			0.981
Yes	4	13	
No	15	48	
**HBsAg**			0.280
Positive	19	54	
Negative	0	7	
**Albumin level, g/L**			>0.999
≤35	3	10	
>35	16	51	
**AFP level, ng/ml**			0.890
≤400	9	30	
>400	10	31	
**CA19-9 level, ng/ml**			0.994
≤37	15	46	
>37	4	15	
**Child-Pugh grade**			>0.999
A	17	56	
B	2	5	
**Edmondson grade**			0.847
Ⅰ-Ⅱ	12	40	
Ⅲ-Ⅳ	7	21	
**BCLC stage**			0.187
A-B	11	45	
C	8	16	

^a^Values are n, unless otherwise indicated. Abbreviations: AFP, alpha-fetoprotein; BCLC, Barcelona Clinic Liver Cancer; DPHCC, dual-phenotype hepatocellular carcinoma; HBsAg, hepatitis B surface antigen; HCC, hepatocellular carcinoma.

**Table 3:** Uni- and multivariate analysis to identify predictors of poor recurrence-free survival and overall survival in HCC patients after hepatectomy (n=80).

Variable	Recurrence-free survival						Overall survival				
	Univariate analysis			Multivariate analysis			Univariate analysis			Multivariate analysis	
	HR (95%CI)	P		HR (95%CI)	P		HR (95%CI)	P		HR (95%CI)	P
DPHCC ( yes )	1.682 ( 0.961-2.947 )	0.069					2.048 ( 1.104-3.801 )	0.023		1.205 ( 0.467-2.252 )	0.951
CK19 ( positive )	1.992 ( 1.204-3.296 )	0.007		1.867 ( 1.124-3.102 )	0.016		2.264 ( 1.263-4.056 )	0.006		2.213 ( 1.070-4.580 )	0.032
Age ( >55 years )	0.771 ( 0.401-1.479 )	0.443					0.660 ( 0.295-1.476 )	0.312			
Liver cirrhosis ( yes )	1.134 ( 0.675-1.906 )	0.635					1.298 ( 0.720-2.338 )	0.385			
BCLC stage ( C )	1.631 ( 0.963-2.762 )	0.069					1.947 ( 1.078-3.514 )	0.027		2.061 ( 1.093-3.888 )	0.025
Tumor size ( >5 cm )	1.353 ( 0.816-2.242 )	0.242					1.723 ( 0.942-3.153 )	0.078			
Multiple nodules	2.158 ( 1.266-3.767 )	0.005		1.868 ( 1.089-3.205 )	0.023		1.961 ( 1.095-3.511 )	0.024		1.395 ( 0.734-2.650 )	0.310
Capsule ( incomplete)	1.001 ( 0.598-1.671 )	0.999					1.332 ( 0.743-2.388 )	0.336			
Tumor thrombi ( yes )	1.115 ( 0.615-2.022 )	0.719					1.283 ( 0.653-2.520 )	0.470			
HBsAg ( positive )	4.638 ( 1.130-19.026 )	0.033		3.628 ( 0.875-15.040 )	0.076		5.872 ( 0.809-42.600 )	0.080			
Albumin level ( <35g/L )	1.842 ( 0.286-3.497 )	0.063					2.053 ( 1.013-1.160 )	0.046		1.376 ( 0.628-3.012 )	0.425
AFP level ( >400 ng/ml )	1.373 ( 0.836-2.254 )	0.210					1.385 ( 0.779-2.462 )	0.268			
CA19-9 level ( >37 ng/ml )	1.982 ( 1.142-3.441 )	0.015		1.655 ( 0.946-2.896 )	0.078		2.101 ( 1.145-3.855 )	0.017		2.222 ( 1.164-4.242 )	0.016
Edmondson grade ( Ⅲ-Ⅳ )	1.637 ( 0.985-2.721 )	0.057					1.395 ( 0.779-2.499 )	0.263			
Child-Pugh grade ( B )	1.186 ( 0.511-2.756 )	0.691					1.080 ( 0.388-3.012 )	0.882			

Abbreviations: AFP, alpha-fetoprotein; BCLC, Barcelona Clinic Liver Cancer; DPHCC, dual-phenotype hepatocellular carcinoma; HBsAg, hepatitis B surface antigen; HCC, hepatocellular carcinoma; HR, hazard ratio

## Abbreviations

DPHCC: dual-phenotype hepatocellular carcinoma
CSCs: cancer stem cells
HCC: Hepatocellular carcinoma
AASLD: American Association for the Study of Liver Diseases
BCLC: Barcelona Clinic Liver Cancer
CA19-9: carbohydrate antigen 19-9
CK19: cytokeratin 19
Hep Par 1: hepatocyte-specific antigen
HR: hazard ratio
CI: Confidence Interval
OS: overall survival
RFS: recurrence-free survival
IL-8: interleukin-8
CD90: cluster of differentiation 90
EPCAM: Epithelial cell adhesion molecule
CD133: cluster of differentiation 133
AFP: alpha-fetoprotein
HBsAg: hepatitis B surface antigen
