# Supplementary material for: Overexpression of Epcam and CD133 Correlates with Poor Prognosis in Dual-phenotype Hepatocellular Carcinoma
Source: J Cancer. 2020 Mar 5;11(11):3400–6. doi: 10.7150/jca.41090 (PMC7097958; doi:10.7150/jca.41090)
Supplement: Supplementary file 1 — Supplementary methods, results, and figure. [file jcav11p3400s1.pdf]

Methods

Bioinformatics

For preliminary verification of experimental results，transcriptome data of 374 HCC patients was extracted in The Cancer Genome Atlas (TCGA) database. 374 cases of HCC were divided into high expression group (top 15% of expression) and low expression group (85% lower expression) according to the expression level of gene KRT19 (target gene of CK19). R statistical and GraphPad Prism software were applied to estimate differentially expression of CD133, THY1 (CD90), and EpCAM between high expression group and low expression group.

Results

Bioinformatics validation of CSC marker expression

Levels of CD90 (THY1), CD133 and EpCAM expression were analyzed from 374 patients with DPHCC which were divided into high expression group and low expression group (Figure 3). High expression group was associated with significantly higher levels of EpCAM (P < 0.001), CD133 (P < 0.001) and CD90 (P =0.0015).

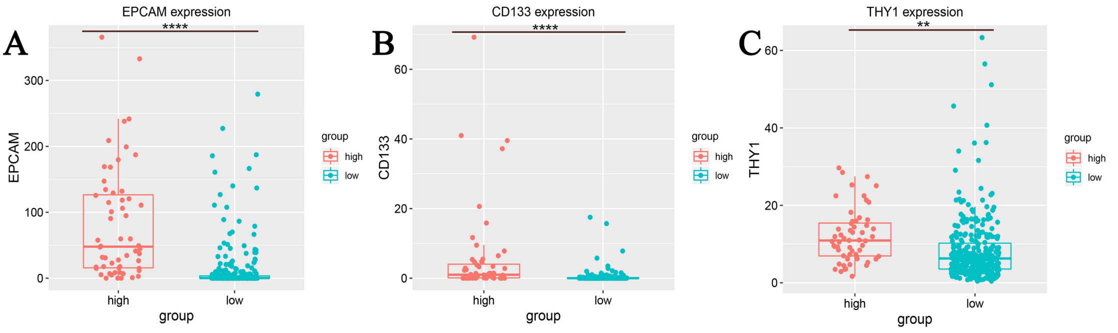

Fig. S1 Differentially expressed CSCs genes between high expression of KRT19 in HCC and low expression of KRT19 in HCC. EpCAM, CD133, and CD90 (THY1) gene expression were significantly up-regulated in 118 cases of high KRT19 expression HCC and 256 cases of low KRT19 expression HCC.
